# Development and Validation of a Nomogram Based on Geriatric Nutritional Risk Index to Predict Surgical Site Infection Among Gynecologic Oncology Patients

**DOI:** 10.3389/fnut.2022.864761

**Published:** 2022-04-27

**Authors:** Zhihui Chen, Mingchen Zhong, Ziqin Xu, Qing Ye, Wenwen Xie, Shengchun Gao, Le Chen, Lidan Qiu, Jiaru Jiang, Hongmei Wu, Xiuyang Li, Haihong Wang

**Affiliations:** ^1^Department of Infection Control, Wenzhou People’s Hospital, Wenzhou, China; ^2^Department of Epidemiology and Biostatistics, Center for Clinical Big Data and Statistic, Second Affiliated Hospital, Medicine College, Zhejiang University, Hangzhou, China; ^3^Scientific Research Center, Wenzhou People’s Hospital, Wenzhou, China; ^4^Xinglin Information Technology Company, Hangzhou, China; ^5^Department of Gynecology, Wenzhou People’s Hospital, Wenzhou, China

**Keywords:** geriatric nutritional risk index, gynecologic oncology, surgical site infection, infection prevention, nomogram, prediction model

## Abstract

**Background:**

The geriatric nutritional risk index (GNRI) is a commonly used method to assess nutritional risk for predicting potential surgical site infections (SSI) in cancer patients. This study aims to create and verify a simple nomogram and a dynamic web-based calculator for predicting the risk of SSI among gynecologic oncology patients.

**Methods:**

A retrospective evaluation was conducted on patients who were admitted into a tertiary hospital in China with confirmed diagnosis of gynecologic cancer between 01 August 2017 and 30 November 2021. A two-piecewise linear regression model with a smoothing function was used to investigate the non-linear association between GNRI and SSI to determine the ideal cut-off point. Three models were developed on the basis of different variables to predict SSI in gynecologic oncology patients. Through a nomogram the concordance index (C-index), the Akaike information criterion (AIC), and the integrated discrimination index (IDI) were used to determine the final model. Finally, the performance of the nomogram was validated using the 1,000-bootstrap resamples method and analyzed using C-index, GiViTI calibration belts, and decision curve. Also, a user-friendly dynamic web-based calculator was developed.

**Results:**

A total of 1,221 patients were included in the analysis. A non-linear association could be observed between GNRI and SSI risk with a GNRI cut-off value of 101.7. After adding GNRI to Model 2 (which comprised Morse Fall Scale score, preoperative length of stay, operation time, and estimated blood loss), the AIC value decreased, the C-index value increased and IDI increased significantly. The nomogram C-index in the development cohort and internal validation cohort demonstrates a moderate-high degree of discrimination. The GiViTI calibrated belt showed a good agreement between the observed and predicted probabilities of SSI. The decision curve validates the clinical feasibility of the nomogram with a threshold value between 0 and 49%.

**Conclusion:**

The GNRI cut-off value of 101.7 allowed for appropriate stratification of patients into distinct SSI risk groups. This study found that including GNRI in the above nomogram (Model 2) would enhance its potential to predict SSI in gynecologic oncology patients.

## Introduction

With an incidence rate of approximately 5–35% following a gynecologic oncology surgery, surgical site infections (SSIs) are considered one of the most prevalent postoperative complications reported among patients globally (1–4). Also considered as one of the primary etiological factors that leads to postoperative nosocomial infections, SSI will bring about an increased morbidity and mortality rate as well as prolonged hospitalization, and higher healthcare expenditures (5–7). Therefore, it is important to identify patients highly susceptible to SSI.

SSI is the outcome of a complex combination of factors relating to the patient, environment, and to the surgery itself (6). Recent studies have also shown and elucidated that nutritional status is also considered a critical factor that is closely associated with the development of SSI in gynecologic oncology patients (8, 9). This suggests that the assessment of malnutrition could provide clinically valuable information regarding the prediction of SSI in gynecologic oncology patients. The Geriatric Nutritional Risk Index (GNRI) is one of a few established scoring systems for measuring the severity of malnutrition in clinical settings. The GNRI calculation takes into account the serum albumin level, current weight, and optimum body weight based on gender and height, all of which are accessible to most patients prior to therapy (10). GNRI is a simple-to-calculate technique that is sensitive to detecting malnutrition (11–13) and has only recently gained popularity in assessing a patient’s nutritional status and in predicting the risk of SSI in cancer patients (14, 15). Some studies have used the GNRI in conjunction with other traditional indicators to predict the development of SSI in gynecologic oncology patients. In combination with the usage of a nomogram as a means of visualization of a mathematical model, may prove beneficial as it incorporates many critical variables rather than analyzing individual risk factors (16). This method provides each patient with personalized, evidence-based, and highly accurate risk estimations that are presented intuitively.

The primary objectives of this study are to (1) investigate potential associations between the GNRI and the risk of SSI in gynecologic oncology patients; (2) investigate the predictive value of including the GNRI in the study models; (3) to develop and validate a nutrition-based nomogram that incorporates the GNRI and clinical risk factors to predict SSI in gynecologic oncology patients.

## Materials and Methods

### Study Design and Participants

**FIGURE 1:**
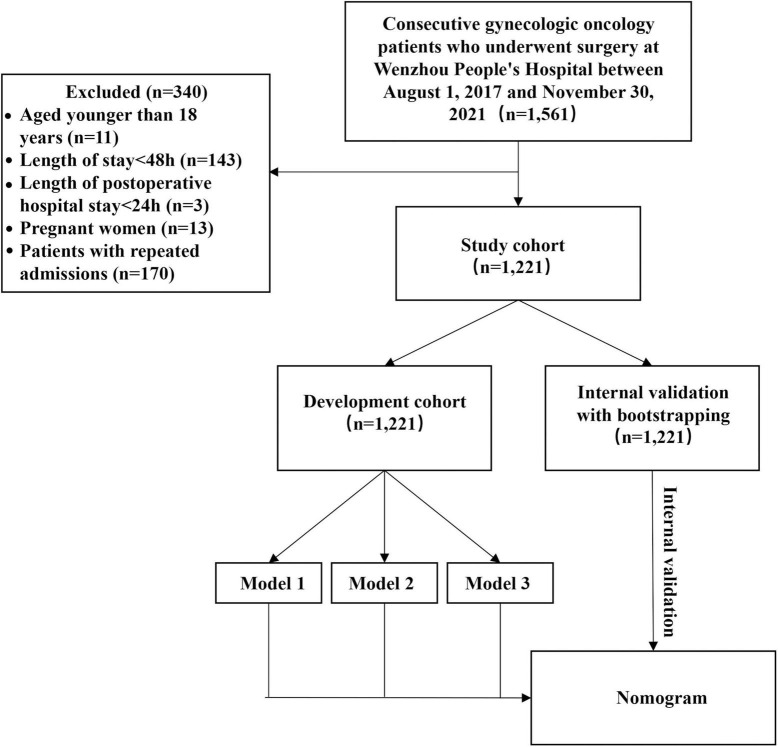
Flow chart of the study population.

To establish the development cohort herein, a total of 1,561 consecutive gynecologic oncology patients who underwent surgery at Wenzhou People’s Hospital between 01 August 2017 and 30 November 2021, were retrospectively evaluated (Figure 1). The following are the inclusion criteria: medically confirmed diagnoses of gynecological (cervix, corpus, ovarian/tubal/peritoneal, vulva, or vaginal) cancer; patients who have received surgery and postoperative care at our institution. Patients under the age of 18 years, patients with a length of stay of less than 48 h, patients who were postoperatively hospitalized for less than 24 h, and pregnant women were excluded from this analysis. For patients admitted more than once during the study period, only the first admission was analyzed. The entirety of this study was approved by the Ethics Review Committee of Wenzhou People’s Hospital (approval no. KY-2022-010). Waiver of informed consent was granted due to the retrospective nature of the study.

### Candidate Predictors

Expert judgment, extensive literature studies, and the availability of clinical data were used to select all possible candidate predictors for potential operative site infections in gynecologic oncology patients (5–9). The following candidate predictors were included in the study: general information [age, body mass index (BMI), season of admission, surgical history in recent 3 months, comorbidities, and the age-adjusted Charlson Comorbidity Index (aCCI)], preoperative variables [FIGO stage, ASA class, site of cancer, Barthel Index, Morse Fall Scale (MFS) score, preoperative steroid use, laboratory values, preoperative hair removal, preoperative length of stay (LOS), antibiotic prophylaxis within 0.5–1 h before operation, and the modified surgical complexity score (MSCS)], and intraoperative variables (surgical approach, operative time, estimated blood loss, blood transfusion, emergent surgery, and NNIS index). Furthermore, the GNRI was also included as a candidate predictor.

Preoperative peripheral blood samples were drawn from patients no more than 7 days prior to surgery. The comorbidity burden was assessed using aCCI (17). Furthermore, the MSCS was used to classify the extent of surgery into two groups (mild and moderate/severe) with respect to the number and complexity of each surgical procedure (5). According to the US National Nosocomial Infection Surveillance System (NNIS), the risk index for nosocomial infections is a coherent additive scale that takes into account patient variability in underlying illness severity, surgical wound contamination level, and surgical process complexity (as measured by surgical procedure duration) (18, 19). This risk index is validated to adequately stratify the risk of SSI in some specific surgeries (20). The GNRI was calculated using the following formulas: GNRI = (1.519 × serum albumin, g/L) + 41.7 × (current body weight [kg]/ideal body weight [kg]) (10). Patients’ current body weight was evaluated by competent nurses within 7 days preoperatively following a standardized protocol. The optimal weight for women was determined using the following Lorenz formula: ideal body weight = height (cm) – 100 – ([height (cm) – 150]/2.5) (21). When a patient’s body weight surpasses the optimal weight, the actual body weight/ideal weight ratio was adjusted to 1. Additional information detailing other candidate predictors is listed in Supplementary Table 1.

### Outcome

The SSIs were characterized according to the definitions set by the Centers for Disease Control (22). The SSIs were classified into incisional and organ/space infections. Incisional infections were further subclassified into superficial and deep incisional infections, whereas organ/space infections were further subcategorized into pelvic cellulitis, pelvic abscess, as well as vaginal cuff infections. The detailed criteria for the abovementioned SSIs can be found in Supplementary Table 2. The SSIs data were acquired and analyzed via the Xinglin real-time nosocomial infection monitoring system. In addition, this system is capable of automatically generating infection warning notices in real-time based on the database of different hospitals [Hospital Information System (HIS), Laboratory Information System (LIS), and Anesthesia Information Management System (AIMS)]. Cases of nosocomial infection documented within the Xinglin system, such as SSIs, must be confirmed after verification by a clinician and a senior infection control practitioner, respectively. Inconsistent opinions were resolved through extensive discussions.

### Statistical Analysis

This study follows the guidelines outlined in the Transparent Reporting of a Multivariable Prediction Model for Individual Prognosis or Diagnosis (TRIPOD) statement (23). To compare categorical variables [summarized as number (%)], the Chi-square test or Fisher’s exact tests were used where applicable. Two-sample Student *t*-test or Mann-Whitney *U* test was employed to compare continuous variables expressed as mean ± standard deviation (SD) or median [interquartile range (IQR)]. All statistical analyses were analyzed using the R software (version 3.6.3)^1^ and a two-tailed *P*-value of ≤ 0.05 was considered to be statistically significant.

#### Missing Data

Prior to data analysis, the distribution of data and missing values for all predictors were examined. The percentage of missing data varied between 0 and 16.5% for each candidate predictor variable. Furthermore, to maximize statistical power and decrease selection bias caused by missing data, we performed multiple imputation with the Multivariate Imputations by Chained Equation (MICE) package in R (Supplementary Table 3) (24).

#### Sample Size Calculation

Based on clinical factors such as MFS score, preoperative LOS, operative time, estimated blood loss, and GNRI, we established a model capable of predicting SSIs in gynecological surgery. The incidence of operative site infection in gynecological surgery is estimated to be 5.3% in this model. The multivariable logistic model included five predictor parameters and had a C statistic of 0.77. We performed a power analysis using the formula developed by Riley et al. (25) and obtained a required minimum sample size for the development of a new model consisting of 851 patients, 46 events (assuming an outcome prevalence = 0.053), and an EPP of 9.02.

#### The Cut-Off Point for Continuous Variables

All participants were divided into three groups using BMI for Chinese men and women as the criteria (26): underweight (BMI < 18.5 kg/m^2^), normal weight (BMI 18.5–24.0 kg/m^2^), and overweight/obese (BMI ≥ 24 kg/m^2^). To categorize the patients, the cut-off point for laboratory values was determined as the upper or lower limit of the normal value. The cut-off point for glucose, albumin, ALT, total bilirubin, platelet count, hematocrit, TLC, WBC was 110 mg/dL, 3.0 g/dL, 40 U/L, 1.1 mg/dL, 350 × 10^9^/L, 36%, 0.8 × 10^9^ /L, and 10 × 10^9^ /L, respectively. The two-piecewise linear regression model with a smoothing function was performed using the GAM package in R to investigate the non-linear relationship between GNRI, preoperative LOS, operative time, estimated blood loss, and SSI. The cut-off point was determined through trial and error, which included selecting turning points within a pre-defined interval and then selecting the ideal cut-off point that produced the highest model likelihood. Furthermore, we ran a log-likelihood ratio test to compare the one-line linear regression model and the two-piecewise linear model.

#### Variables Selection and Model Development

On the basis of different inclusions of variables, we have developed three models to predict the potential operative site infections in a gynecologic oncology surgery:(a) candidate predictor for Model 1 was limited to only the NNIS risk index; (b) candidate predictors for Model 2 included all factors indicated in Table 1 except GNRI; (c) candidate predictors for Model 3 included all variables listed in Table 1. To select the most optimal predictive features from the candidate predictors, the least absolute shrinkage and selection operator (LASSO) method was performed using the R package glmnet (9). This method is well-suited for reducing large datasets and reducing the likelihood of collinearity between the variables obtained from the same subject. The 10-fold cross-validation method was also used to determine the ideal value of the penalty parameter λ. Furthermore, the variables with non-zero coefficients in the fitted LASSO model were considered significant predictors. Candidate predictors selected through the LASSO method were used to construct a multivariate logistic regression model, and the Akaike Information Criterion (AIC) was referenced to find the optimal prediction model (12). The above analysis was performed using function glm in the “stats” package and function stepAIC in the “MASS” package. Finally, the Harrell concordance index (C-index), AIC, and the integrated discrimination index (IDI) were used to determine the final model (13, 14).

**TABLE 1:** Baseline characteristics of development cohort after imputation.

Characteristic	Non-SSI (*n* = 1,156)	SSI (*n* = 65)	*P*
**General information**			
Age (years), median (IQR)	50 (18)	56 (18)	0.010
BMI (kg/m^2^), *n* (%)			0.786
<18.5	44 (3.8)	2 (3.1)	
18.5–24.0	662 (57.3)	40 (61.5)	
≥24	450 (38.9)	23 (35.4)	
Season of admission, *n* (%)			0.652
Spring	218 (18.9)	11 (16.9)	
Summer	259 (22.4)	12 (18.5)	
Fall	383 (33.1)	21 (32.3)	
Winter	296 (25.6)	21 (32.3)	
Surgical history in recent 3 months, *n* (%)	45 (3.9)	4 (6.2)	0.366
Comorbidities			
Hypertension, *n* (%)	290 (25.1)	26 (40.0)	0.008
Diabetes, *n* (%)	135 (11.7)	14 (21.5)	0.018
Coronary artery disease, *n* (%)	20 (1.7)	3 (4.6)	0.096
COPD/emphysema, *n* (%)	13 (1.1)	2 (3.1)	0.164
Moderate or severe renal disease, *n* (%)	32 (2.8)	3 (4.6)	0.385
Liver disease, *n* (%)	275 (23.8)	23 (35.4)	0.034
Bacterial vaginosis, *n* (%)	43 (3.7)	1 (1.5)	0.359
aCCI (points), median (IQR)	1 (4)	3 (5)	0.002
**Preoperative variables**			
FIGO stage ≥ III, *n* (%)	92 (8.0)	6 (9.2)	0.713
ASA class ≥ III, *n* (%)	58 (5.0)	4 (6.2)	0.685
Site of cancer, *n* (%)			0.109
Cervix	792 (68.5)	43 (66.2)	
Ovary/Fallopia	144 (12.5)	4 (6.2)	
Tube/Peritoneum uterus	220 (19.0)	18 (27.7)	
Barthel index, *n* (%)			0.102
Independent	1,076 (93.1)	57 (87.7)	
Partially/Totally dependent	80 (6.9)	8 (12.3)	
MFS score, *n* (%)			0.002
No risk	891 (77.1)	39 (60.0)	
Low/High risk	265 (22.9)	26 (40.0)	
Preoperative steroid use, *n* (%)	13 (1.1)	1 (1.5)	0.760
Laboratory values			
Glucose > 110 mg/dL, *n* (%)	891 (17.5)	14 (21.5)	0.403
Albumin ≤ 3.0 g/dL, *n* (%)	86 (7.4)	6 (9.2)	0.594
ALT > 40 U/L, *n* (%)	88 (7.6)	5 (7.7)	0.981
Total bilirubin ≥ 1.1 mg/dL, *n* (%)	79 (6.8)	4 (6.2)	0.832
Platelet count > 350 × 10^9^/L, *n* (%)	47 (4.1)	6 (9.2)	0.047
Hematocrit < 36%, *n* (%)	377 (32.6)	24 (36.9)	0.472
TLC < 0.8 × 10^9^ /L, *n* (%)	38 (3.3)	2 (3.1)	0.926
WBC > 10 × 10^9^ /L, *n* (%)	48 (4.2)	5 (7.7)	0.173
Preoperative hair removal, *n* (%)	1,119 (96.8)	64 (98.5)	0.453
Preoperative LOS (d), median (IQR)	2.0 (2.0)	4.0 (5.0)	<0.001
Antibiotic prophylaxis within 0.5–1 h before operation, *n* (%)	815 (70.5)	53 (81.5)	0.056
MSCS>2, *n* (%)	40 (3.5)	3 (4.6)	0.623
**Intraoperative variables**			
Surgical approach, *n* (%)			0.417
Laparotomy	611 (52.9)	31 (47.7)	
Laparoscopy	545 (47.1)	34 (52.3)	
Operative time (min), median (IQR)	88.0 (130.5)	180.0 (152.0)	<0.001
Estimated blood loss (mL), median (IQR)	50.0 (90.0)	100.0 (250.0)	<0.001
Blood transfusion, *n* (%)	55 (4.8)	5 (7.7)	0.287
Emergent surgery, *n* (%)	185 (16.0)	8 (12.3)	0.427
NNIS risk index ≥ 1, *n* (%)	253 (21.9)	33 (50.8)	<0.001
**Nutrition risk screening tools**			
GNRI (points), median (IQR)	102.6 (8.5)	99.9 (6.7)	<0.001

*IQR, interquartile range; SSI, surgical site infection; BMI, body mass index; COPD, chronic obstructive pulmonary disease; aCCI, age-adjusted Charlson comorbidity index; FIGO, International Federation of Gynecology and Obstetrics; ASA, American Society of Anesthesiology; MFS, Morse Fall Scale; ALT, alanine aminotransferase; TLC, total lymphocyte count; WBC, white cell count; LOS, length of stay; MSCS, modified surgical complexity score; NNIS, National Nosocomial Infection Surveillance; GNRI, geriatric nutritional risk index.*

Based on the regression coefficients of the chosen independent variables, a simple nomogram was established by R package “regplot” to predict the SSIs in gynecologic oncology patients.

#### Accuracy and Reliability Evaluation of Prediction Model

In order to obtain an unbiased assessment of model performance, 1,000-bootstrap resamples were used for internal validation (27). Discrimination and calibration were used to describe the model’s prediction performance. The nomogram’s discrimination ability was evaluated using the concordance index (C-index; equal to the area under the receiver operating curve). A value higher than 0.75 indicates relatively good discrimination in the C-index value, which ranges from 0.5 to 1.0. An evaluation of the nomogram’s calibration was carried out by plotting the GiViTI calibration belts (28). The 0.95 confidence band of the calibration curve and calibration test was used to detect the discrepancy between predicted and observed probabilities. The 95% CI did not cross the bisector, indicating a statistically significant deviation from the predicted probabilities. The calibration test (*P*-value > 0.05) suggests that there was no evidence of poor fit of the developed model. In addition, the existing predictors were also evaluated for their accuracy and suitability. A variety of statistical techniques, including the Cook’s distance, the studentized residuals, the variance inflation factor (VIF), and the hat value were used to identify the outliers, collinearity, influential observations, as well as data with high leverage.

#### Clinical Utility

The current study utilizes the decision curve analysis (DCA) to evaluate the clinical efficacy of the established nomogram (29). DCA is a novel method that could be applied to a clinical setting to assess the potential advantages of a risk prediction model, and it is derived using the formula provided below:


$$\mathrm{Net}\mathrm{benefit}=\mathrm{true}\mathrm{positive}\mathrm{rate}-\mathrm{false}\mathrm{positive}\mathrm{rate}\times \frac{p_{t}}{1-p_{t}}$$

where, *p*_*t*_ represents the threshold probability at which the expected benefit of intervention-all-patients is equal to the expected benefit of intervention-none.

## Results

### Characteristics of the Study Cohort

Among the 1,561 cohort participants, a total of 340 patients of which were individuals who were under 18 years of age (11 patients, 0.7%), patients with the length of stay less than 48 h (143 patients, 9.2%), patients who were postoperatively hospitalized for less than 24 h (3 patients, 0.2%), pregnant patients (13 patients, 0.8%), and as to not introduce bias due to hospital readmissions, another 170 patients (10.9%) were excluded from the study. Finally, the final analysis consisted of 1,221 participants (Figure 1). Among them, 65 patients (5.3%) had an SSI within 30 days of gynecologic oncology surgery. Baseline characteristics of the study cohort after imputation in regard to SSI vs. no SSI are listed in Table 1 and Supplementary Table 3.

### Association Between Geriatric Nutritional Risk Index and Surgical Site Infections

**FIGURE 2:**
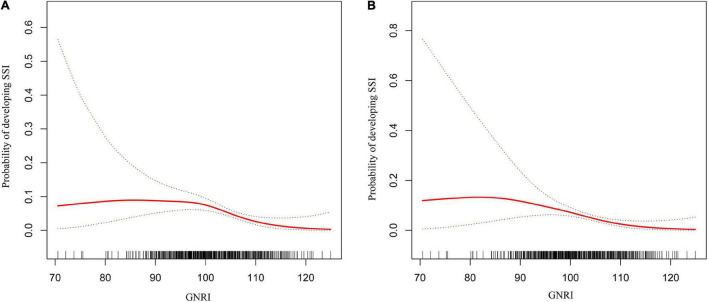
Two-piece piecewise regression and smooth curve-ftting for association between GNRI and the risk of SSI in gynecologic oncology patients. **(A)** The two-piece wise models unadjusted for any variables. **(B)** The two-piece wise models adjusted for age, BMI, season of admission, surgical history in recent 3 months, hypertension, diabetes, coronary artery disease, COPD/emphysema, moderate or severe renal disease, liver disease, bacterial vaginosis, aCCI, FIGO stage, ASA class, site of cancer, Barthel Index, MFS score, preoperative steroid use, glucose, albumin, ALT, total bilirubin, platelet count, hematocrit, TLC, WBC, preoperative hair removal, preoperative LOS, antibiotic prophylaxis within 0.5–1 h before operation, surgical approach, operative time, estimated blood loss, blood transfusion, emergent surgery, NNIS risk index, and GNRI. Note: the red line represents the best-fit line, and the blue lines are the 95% confidence intervals.

Before and after adjusting for the factors that impact the association between GNRI and SSI, the results of the logistic regression analysis both revealed that GNRI remained strongly linked with SSI when examined as continuous variables (Table 2). Furthermore, a smooth curve fitting graph was used before and after all variables were adjusted, and the resultant curve exhibited a two-stage change and a cut-off point (Figures 2A,B). Although the SSI risk is lower when GNRI is higher than the cut-off point, there is no discernible difference when GNRI is lower than the cut-off point. The saturation effects were analyzed based on the curve, and the data indicated that the cut-off point was 101.7 (Table 2).

**TABLE 2:** Cut-off point of GNRI before and after adjustment of the effect modifier.

		GNRI	
		Crude	Adjusted^†^
One-line linear regression model	OR (95% CI)	0.95 (0.92, 0.98)	0.92 (0.87, 0.97)
Two-piecewise linear model	Cut-off point	101.7	101.7
	OR1 (95% CI)	1.01 (0.95, 1.08)	1.00 (0.91, 1.11)
	OR2 (95% CI)	0.83 (0.74, 0.93)	0.83 (0.73, 0.94)
	OR2/OR1 (95% CI)	0.82 (0.71, 0.96)	0.83 (0.69, 1.00)
	Logarithmic likelihood ratio test	0.006	0.036

*OR, odds ratio; CI, confidence interval; GNRI, geriatric nutritional risk index.*

*^†^Adjusted for age, BMI, season of admission, surgical history in recent 3 months, hypertension, diabetes, coronary artery disease, COPD/emphysema, moderate or severe renal disease, liver disease, bacterial vaginosis, aCCI, FIGO stage, ASA class, site of cancer, Barthel Index, MFS score, preoperative steroid use, glucose, albumin, ALT, total bilirubin, platelet count, hematocrit, TLC, WBC, preoperative hair removal, preoperative LOS, antibiotic prophylaxis within 0.5–1 h before operation, surgical approach, operative time, estimated blood loss, blood transfusion, emergent surgery, and NNIS risk index.*

Moreover, we used a smoothing function to investigate the non-linear relationship between additional continuous variables (preoperative LOS, operative time, and estimated blood loss), and SSI in a two-piecewise linear regression model in which the results demonstrated a linear relationship between preoperative LOS and the risk of SSI. In contrast, a non-linear relationship could be seen between the duration of surgery and estimated blood loss with the risk of SSI (Supplementary Figures 1A–C). Based on the saturation effect of the curve analysis, the operating duration and estimated blood loss thresholds were 145 and 40, respectively (Supplementary Table 4).

### Feature Selection

**FIGURE 3:**
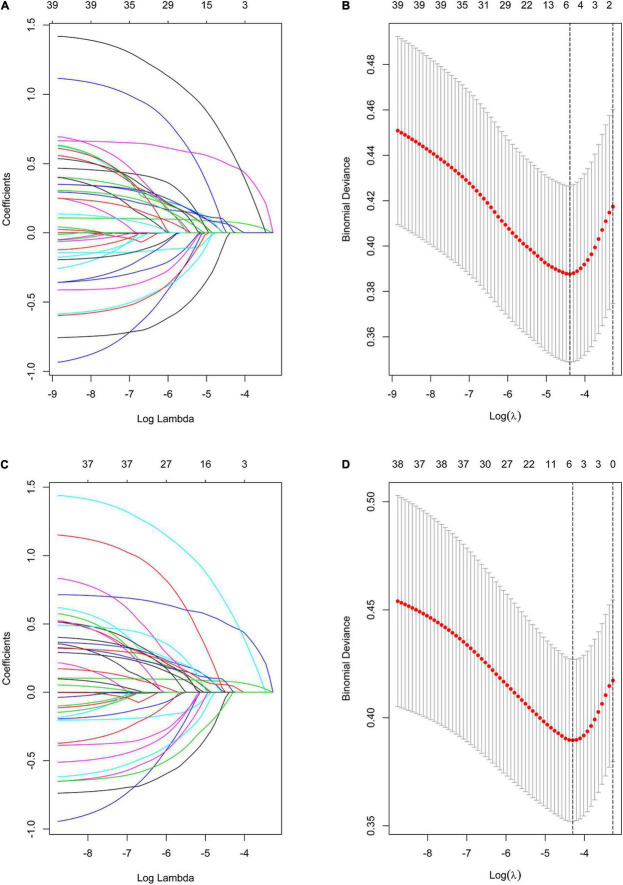
Variable selection using the LASSO binary logistic regression model. **(A)** Profiles of the LASSO coefficients for the 36 candidate variables. **(B)** Optimal penalization coefficient (λ) selection in the LASSO model used 10-fold cross-validation via minimum criteria. **(C)** Profiles of the LASSO coefficients for the 37 (Plus GNRI) candidate variables. **(D)** Optimal penalization coefficient (λ) selection in the LASSO model used 10-fold cross-validation via minimum criteria. Note: the left vertical line represents the minimum error, and the right vertical line represents the one standard error of the minimum criteria (1-SE criterion).

Using the LASSO method, five predictive features with non-zero coefficients (including MFS score, preoperative LOS, operation time, NNIS risk index, and estimated blood loss) were screened from the 36 candidate predictors (Figures 3A,B), and six predictive features with non-zero coefficients (including MFS score, preoperative LOS, operation time, NNIS risk index, estimated blood loss, and GNRI) were screened from the 37 candidate predictors (Figures 3C,D). Besides, the NNIS risk index was excluded in the final constructed multivariate regression model (Model 2 and Model 3) according to the lowest principle of AIC (Table 3).

**TABLE 3:** Prediction effect of the three models.

Intercept and variable	Model 1^†^		Model 2^‡^		Model 3^§^	
	β	Adjusted OR (95% CI)	β	Adjusted OR (95% CI)	β	Adjusted OR(95% CI)
Intercept	−3.34		−4.49	−	−4.49	−
MFS score (Low/High Risk)	−	−	0.50	1.65(0.96–2.78)	0.41	1.51(0.87–2.57)
Preoperative LOS (increase per day)	−	−	0.27	1.32(0.66–2.76)	0.10	1.10(1.02–1.18)
Operative time ≥ 145 min	−	−	0.69	1.99(0.91–4.20)	0.70	2.01(1.14–3.65)
Estimated blood loss ≥ 40 ml	−	−	1.39	4.01(1.79–10.22)	1.31	3.69(1.65–9.42)
NNIS risk index ≥ 1	1.30	3.68(2.22–6.12)	−	−	−	−
GNRI ≥ 101.7	−	−	−	−	−0.52	0.60(0.35–1.00)
		C_1_-index		C_2_-index		C_3_-index
Primary cohort		0.644		0.745		0.770
Internal validation with 1000 bootstrapping		0.644		0.743		0.768
AIC		487.44		468.23		458.99
IDI (95%CI)		7.28% (3.46%–11.09%)*		5.42% (1.80%−9.03%)**		

*LOS, length of stay; NNIS, National Nosocomial Infection Surveillance; GNRI, geriatric nutritional risk index; OR, odds ratio; CI, confidence interval; C-index, concordance index; IDI, net reclassification improvement; AIC, akaike information criterion.*

*^†^Candidate predictor for Model 1 was limited to only the NNIS risk index.*

*^‡^Candidate predictors for Model 2 included all factors indicated in Table 1 except GNRI.*

*^§^Candidate predictors for Model 3 included all variables listed in Table 1.*

*for Model 3 vs Model 1.

**for Model 3 vs Model 2.

Table 3 compares the predictive effectiveness of adding preoperative and intraoperative indexes, as well as the GNRI, to Model 1 (which only comprised the NNIS risk index) in predicting SSI. Model 1 exhibits the lowest C-index and the highest AIC values of 0.644 and 487.44, respectively. In contrast, there were four variables (MFS score, preoperative LOS, operation time, and estimated blood loss) that led to a greater risk of SSI in gynecologic oncology patients in Model 2 based on the LASSO and multivariate logistic regression analysis (Figure 3 and Table 3). The elevated C-index value of 0.745–0.770 as well as a decrease of AIC from 468.23 to 458.99 in the development cohort may be attributable to the addition of GNRI to Model 2 containing the aforementioned four variables. To further evaluate whether the addition of GNRI data into the predictors would improve the risk classification with regard to SSI development, IDI was used. After the addition of another factor into the prediction model, the IDI value significantly improved [Model 3 vs. Model 2: 5.42% (1.80–9.03%)].

### Development of a Nutrition-Based Nomogram

**FIGURE 4:**
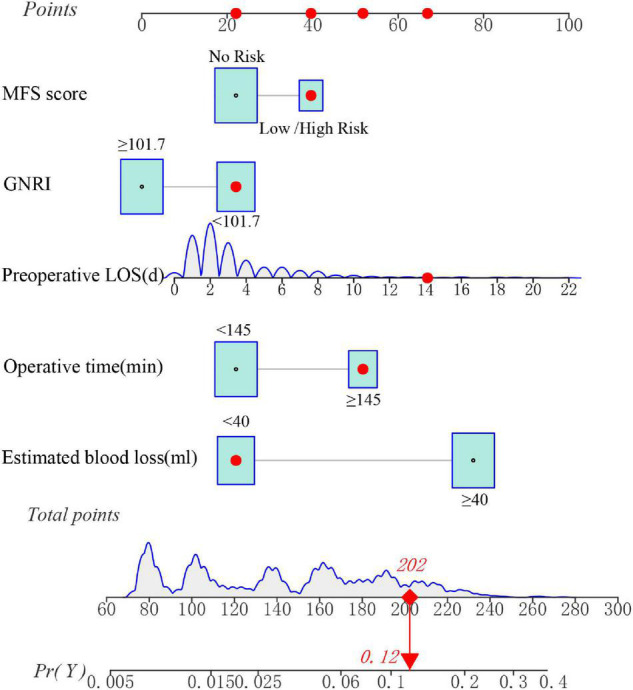
A dynamic nomogram for predicting the risk of SSI in gynecologic oncology patients. Note: the SSI risk nomogram was developed with the MFS score, preoperative LOS, operative time, estimated blood loss, and GNRI as predictors.

A unique nutrition-based nomogram was constructed to predict the likelihood of SSI in gynecologic oncology patients using the five independent variables aforementioned (Figure 4). In addition, for this particular model, a visual and operational dynamic web-based calculator was created by using the R package shiny Through the use of this calculator, users may easily acquire the SSI prediction probability simply by inputting or selecting a variable in the graphical user interface^2^. For instance, the estimated SSI risk was assessed to be approximately 12% (Figure 4 and Supplementary Figure 2) in the dynamic nomogram for SSI in gynecologic oncology patients with MFS score = Low/High Risk (≥ 25 points), preoperative LOS = 14 d, operative time = 160 min, estimated blood loss = 30 ml, and GNRI = 100.9.

### Prediction Model Validation

**FIGURE 5:**
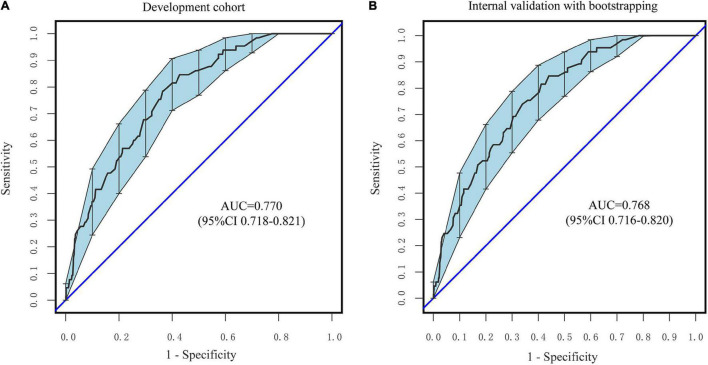
ROC curves of the nomogram. **(A)** The development Cohort. **(B)** The internal validation cohort. The *X*-axis represents the false-positive rate of the risk prediction. The *Y*-axis meant the true-positive rate of the risk prediction.

According to Figures 5A,B, the nomogram C-index in the development cohort was at 0.770 (95% CI: 0.718–0.821) and 0.768 (95% CI: 0.716–0.820) in the internal validation cohort, respectively. Based on these findings, the nomogram demonstrates a moderate to high degree of discrimination.

**FIGURE 6:**
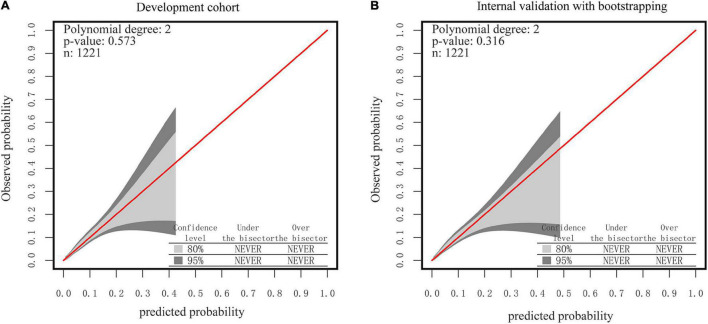
The GiViTI calibration belt for the nomogram. **(A)** The development Cohort. **(B)** The internal validation cohort. Note: the 80%CI and 95%CI calibration belt are plotted, in light and dark gray, respectively. The red diagonal line is the reference line indicating perfect calibration. Note: the red line represents the best-fit line, and the blue lines are 95% confidence intervals.

As seen in Figures 6A,B, the 95% CI region of the calibrated GiViTI belt did not cross the 45-degree diagonal bisector line in either the development or internal validation cohorts (*P* = 0.573, *P* = 0.316, respectively), indicating that the observed and predicted probabilities of SSI in the prediction model were in good agreement. Because the VIF in all predictors was less than 5, no multicollinearity was seen. Moreover, there were also no observable changes and high leverage cases since both Cook’s distances and hat values were no more than 0.1 and 0.2, respectively (Supplementary Figures 3A,B). Taken together, these findings revealed that the nutrition-based nomogram was a good model for predicting SSI in gynecologic oncology patients.

### Clinical Usefulness of the Nomogram

**FIGURE 7:**
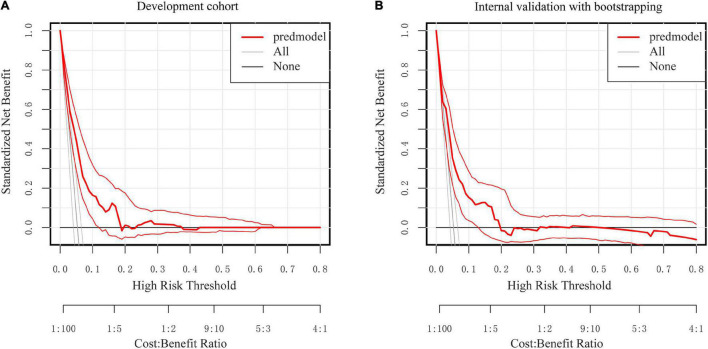
Decision curve analysis for the nomogram. **(A)** The development Cohort. **(B)** The internal validation cohort. The *Y*-axis represents the standardized net benefit. The thick red solid line is the nomogram to predict SSI risk. The thin red solid line represents the 95% credible interval. The black solid line represents the assumption that all patients had no SSI. The gray solid line represents the assumption that all patients had SSI.

According to DCA, using the nomogram to predict SSI risk is more beneficial than using the “intervention-all-patients” or “intervention-none” methods within a range of 0–0.49 (Figures 7A,B).

## Discussion

The rise of SSIs has now become a serious safety problem for gynecologic oncology patients since it not only jeopardizes their health with the increase in mortality rate but will also necessitate much greater treatment costs and longer hospitalization. The ability to accurately identify individuals with a high risk of infection and alter therapy measures appropriately offers an improved preventative approach that may improve health outcomes. As a result, it is imperative that those at greater risk should be promptly identified.

Using the five independent predictors listed above (with reference to Results: Development of a nutrition-based nomogram), we developed a simple yet efficient nomogram to predict surgical site infection in gynecologic oncology patients to aid in medical decision-making. In both the training and validation cohorts, the nomogram demonstrated excellent calibration and discrimination performance. Furthermore, as compared to Model 1 (only NNIS risk index included) and Model 2 (without GNRI), the nomogram based on GNRI demonstrated higher prediction reliability, accuracy, and net benefit. This nomogram can assist physicians in making more precise clinical judgments. By only incorporating these five factors in our nomogram and accounting for varied proportions of those variables, we were able to simply and effortlessly provide an accurate forecast of a patient’s likelihood of acquiring SSI in a systematic and thorough manner. In addition, the serum albumin level, height, as well as the patient’s body weight that make up the GNRI in this study, were routinely assessed before surgery and did not necessitate a special examination, highlighting its feasibility in clinical settings. The GNRI was initially designed to assess malnutrition, as well as its associated morbidity and mortality in elderly patients (10). Numerous studies have shown that GNRI is a suitable predictor for the development of SSI in individuals with various forms of cancer (10, 30, 31). However, the optimal cut-off point value of GNRI for predicting SSIs in cancer patients is still unknown. In a previous study, Sasaki et al. (30) utilized the ROC curve to derive a GNRI cut-off point value of 98 to predict potential prognosis and postoperative complications of elderly patients with colorectal cancer. A similar approach was used to obtain a GNRI cut-off point value of 94 by Funamizu et al. (31) in their study to predict the post-pancreatoduodenectomy development of SSI. In this study, we investigated the non-linear association between GNRI and SSI in gynecologic oncology patients using a two-piecewise linear regression model fitted with a smoothing function and ultimately found the ideal cut-off point value for GNRI was 101.7. There are several possible reasons for the uncertainty surrounding the GNRI cut-off point: Firstly, different malignant tumors exhibit unique biological characteristics; Secondly, the limited number of patients may lead to the bias of the best cut-off value; and finally because there are numerous statistical methods for calculating the best cut-off point value, different cut-off points may be chosen in different studies. For instance, when defining the cut-off value, the ROC curve cannot correct the potential confounding factors within the model, but the two-piecewise linear regression can fully adjust the confounding factors and explain the non-linear connection between the variables (30, 32, 33).

The Morse Fall Scale score was also included in our nomogram. This score is often regarded as a significant indicator of frailty (34–36). Additionally, this study demonstrated that surgical time might independently predict the development of SSI in patients with gynecologic cancer, with 145 min serving as the cut-off value for categorization. It has been proven that a prolonged surgical duration increases the incidence of SSI (37). Also, many prior studies have found a link between estimated blood loss and the risk of SSI (38, 39). According to our study, the development of SSI in gynecologic oncology patients may also be determined using predicted blood loss as an independent variable, with the classification cut-off value set as 40 ml. In addition, the preoperative length of stay was also regarded as an independent predictor of the development of SSI in gynecological oncology patients and was included in the model in accordance with a linear relationship. It should also be noted that, although the NNIS risk index was retained in the model after LASSO dimension reduction using, this factor was excluded in the final constructed multivariate regression model according to the lowest principle of AIC. This further establishes that the NNIS risk index is unsuitable for stratifying the risk of surgical site infection across all types of surgery (19, 40). Mahdi et al. (5) found that the risk of developing SSI for endometrial cancer was 0.8 times higher than that for other types of gynecologic oncology patients in the laparotomy group by multivariate analysis (OR, 1.8; 95% CI,1.2–2.6; *P* = 0.02). However, by multivariate analysis, the above association was not observed in the laparoscopic surgery group (5). Our study showed that the site of cancer was not an independent predictor for SSI in gynecologic oncology patients, which may be attributed to the inclusion of a subset of laparoscopic surgery patients in our cohort.

Additionally, a dynamic web-based calculator was developed to facilitate the use of the nomogram (see text footnote 2). For this particular model, users may easily acquire the SSI prediction probability simply by inputting or selecting a variable in the graphical user interface. Consequently. healthcare providers may conduct a preliminary assessment of the risk of development of SSI in gynecologic oncology individuals and closely monitor those who are more susceptible. A subset of people who would benefit the most from more regular examinations is the high-risk population. In addition, interventions (e.g., nutritional interventions) can be taken more aggressively in high-risk individuals.

There are, however, several limitations that must be taken into account. To begin, this is a retrospective single-center research, which inevitably has limitations due to its methodology and sample size. Therefore, further research is needed to externally validate the suggested nomogram despite the fact that we employed 1,000-bootstrap resamples for internal validation. Secondly, knowing that the cause of SSI is multifactorial, our research was primarily concerned with patient-related and surgical variables, excluding other elements like the operating room setting. Thirdly, there were too many missing data (≥50%) or too few positives in this study to incorporate previously established risk factors for SSI, such as current smokers and intraoperative hypothermia. Fourthly, while each case of SSI was thoroughly evaluated and co-confirmed by a physician and a senior infection control practitioner to avoid misclassification bias in the current investigation, misdiagnosis and missed diagnoses could still have occurred. Finally, even though various assessment tools, such as Barthel Index, Morse Fall Scale, weight, and height, were evaluated by competent nurses in our hospital, we could not rule out the possibility of measurement bias as a result of the screening being performed by different nurses.

## Conclusion

The current research investigates the use of preoperative GNRI as an independent predictor of SSI in gynecologic oncology patients. We found that a cut-off value of 101.7 for the GNRI allowed for appropriate stratification of patients into distinct SSI risk groups. Moreover, we established and validated a nomogram based on the GNRI to estimate the risk of SSI in gynecologic oncology patients, and proved that incorporating the GNRI into the model may further increase its prediction power.

## Data Availability Statement

The raw data supporting the conclusions of this article will be made available by the authors, without undue reservation.

## Ethics Statement

The studies involving human participants were reviewed and approved by the Ethics Review Committee of Wenzhou People’s Hospital. Written informed consent for participation was not required for this study in accordance with the National Legislation and the Institutional Requirements.

## Author Contributions

ZC and MZ analyzed the data and drafted the manuscript. ZX, QY, WX, SG, LC, LQ, JJ, and HWu collected and analyzed the data. XL and HWa conceived and designed the study. All authors read and approved the final manuscript.

## Conflict of Interest

The authors declare that the research was conducted in the absence of any commercial or financial relationships that could be construed as a potential conflict of interest.

## Publisher’s Note

All claims expressed in this article are solely those of the authors and do not necessarily represent those of their affiliated organizations, or those of the publisher, the editors and the reviewers. Any product that may be evaluated in this article, or claim that may be made by its manufacturer, is not guaranteed or endorsed by the publisher.

## Footnotes

1
https://www.R-project.org

2
https://ssi-prediction.shinyapps.io/DynNomapp/

## Supplementary Material

Supplementary Figure 1 Two-piece piecewise regression and smooth curve-ftting to analyze the association between preoperative LOS, operative time, estimated blood loss and the risk of SSI in gynecologic oncology patients. **(A)** The association between preoperative LOS and the risk of SSI. **(B)** The association between operative time and the risk of SSI. **(C)** The association between estimated blood loss and the risk of SSI. All variables (preoperative LOS, operative time, estimated blood loss) in Table 1 were adjusted except self.

Supplementary Figure 2 Screenshot of the online program used for the prediction of SSI risk.

Supplementary Figure 3 Diagnostic plots of accuracy and suitability of existing predictors. **(A)** The Cook’s distance, the studentized residuals, and the hat value of the model. **(B)** The variance inflation factor of the model.
