# Supplementary figures and images for: Development and Validation of a Nomogram Based on Geriatric Nutritional Risk Index to Predict Surgical Site Infection Among Gynecologic Oncology Patients
Source: Front Nutr. 2022 Apr 27;9:864761. doi: 10.3389/fnut.2022.864761 (PMC9097080; doi:10.3389/fnut.2022.864761)

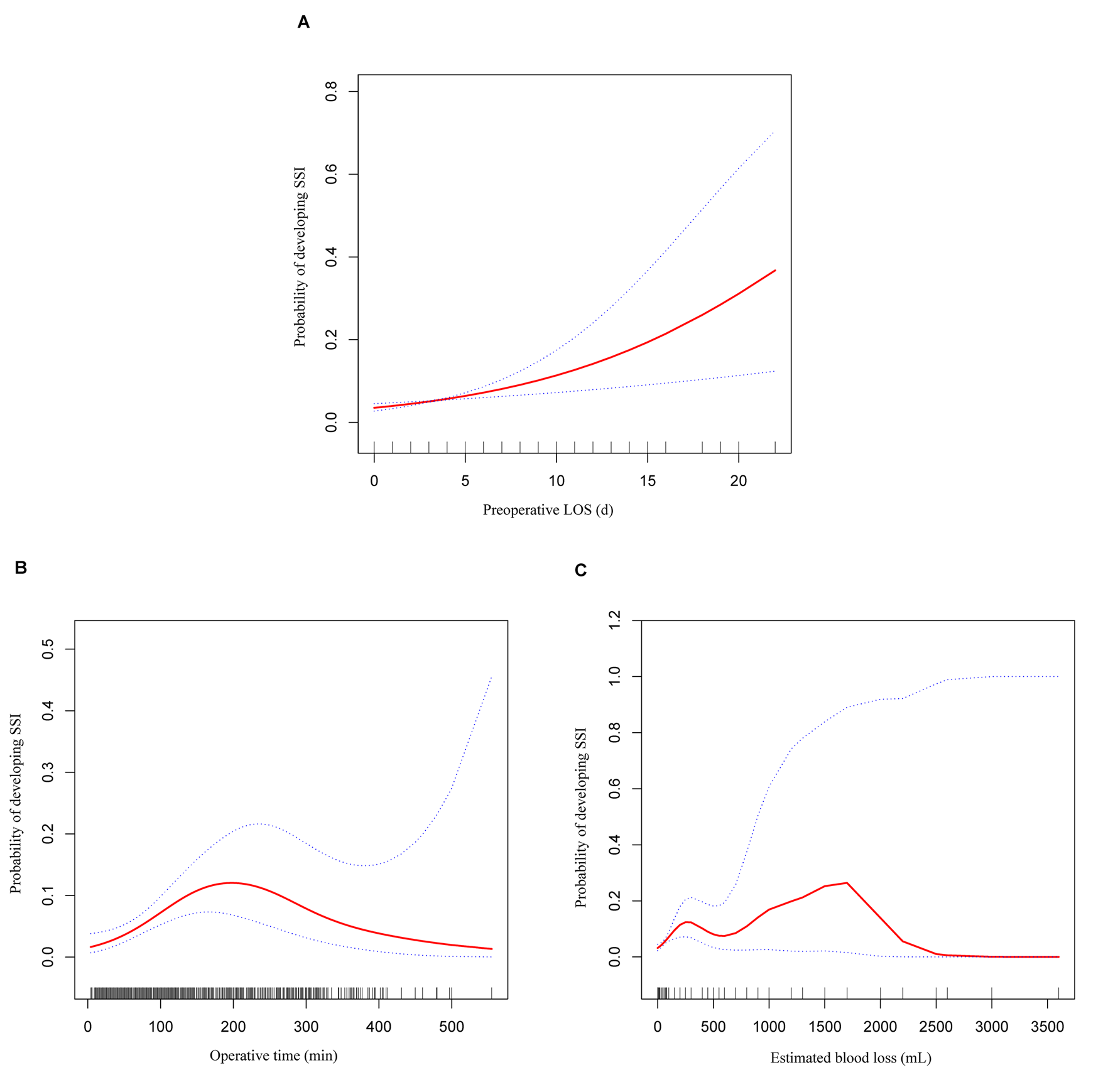

Supplement: Supplementary Figure 1 — Two-piece piecewise regression and smooth curve-ftting to analyze the association between preoperative LOS, operative time, estimated blood loss and the risk of SSI in gynecologic oncology patients. (A) The association between preoperative LOS and the risk of SSI. (B) The association between operative time and the risk of SSI. (C) The association between estimated blood loss and the risk of SSI. All variables (preoperative LOS, operative time, estimated blood loss) in Table 1 were adjusted except self. [file Image_1.TIF]

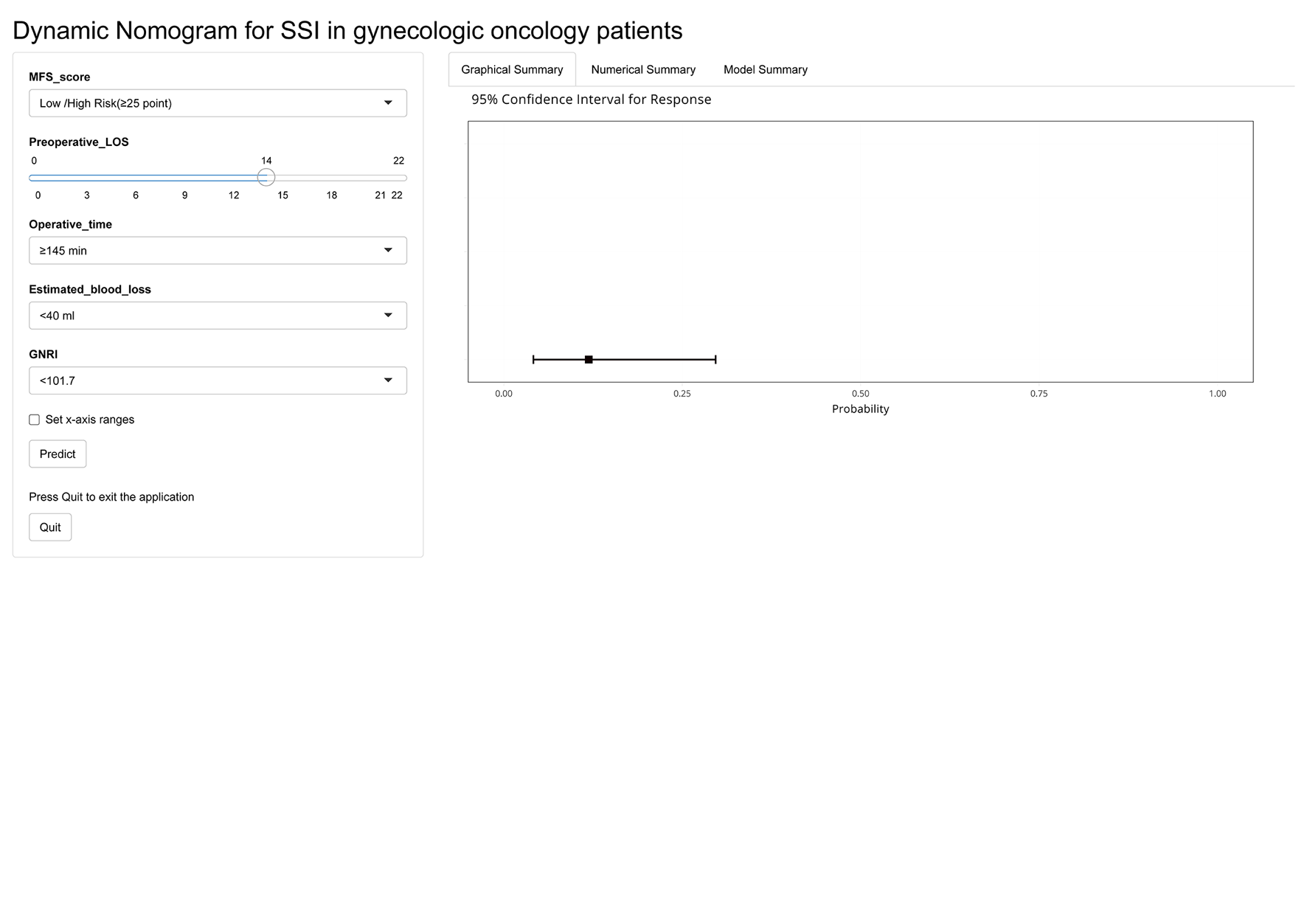

Supplement: Supplementary Figure 2 — Screenshot of the online program used for the prediction of SSI risk. [file Image_2.TIF]

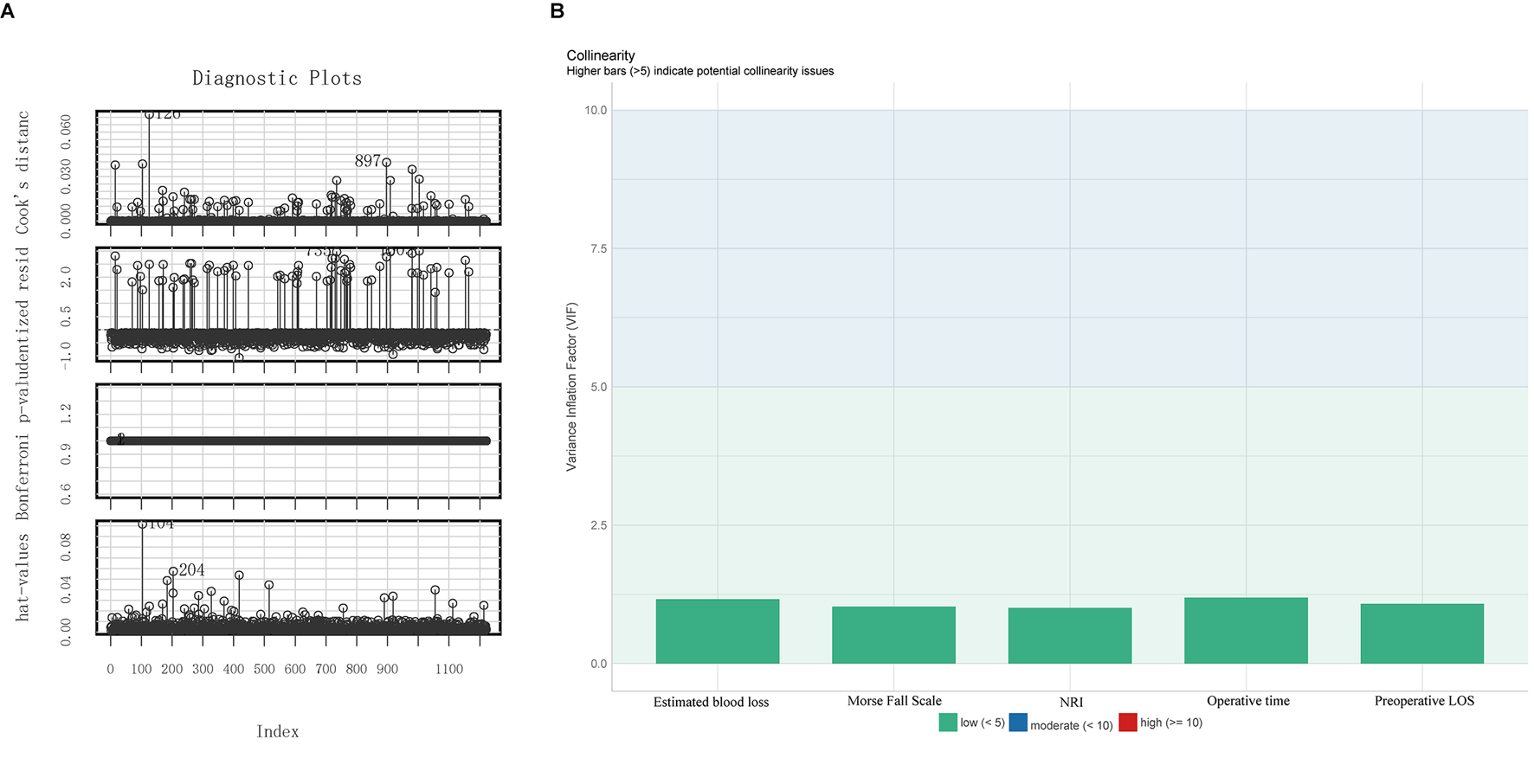

Supplement: Supplementary Figure 3 — Diagnostic plots of accuracy and suitability of existing predictors. (A) The Cook’s distance, the studentized residuals, and the hat value of the model. (B) The variance inflation factor of the model. [file Image_3.TIF]
