# Supplementary material for: Development and Validation of a Nomogram Based on Geriatric Nutritional Risk Index to Predict Surgical Site Infection Among Gynecologic Oncology Patients
Source: Front Nutr. 2022 Apr 27;9:864761. doi: 10.3389/fnut.2022.864761 (PMC9097080; doi:10.3389/fnut.2022.864761)
Supplement: Supplementary file 4 [file Table_1.DOC]

**Table S1. Definition of candidate predictors in development cohort.**

| **Variables** | **Method of assessment and grading** | **Ref.** |
| --- | --- | --- |
| **General information** |  |  |
| Age | Assessed at surgery | in units of year |
| BMI | Defined as the body mass divided by the square of the body height | in units of kg/m^2^ |
| Season of admission | Season of admission | Spring |
| Comorbidities |  |  |
| Hypertension | Admission diagnoses | No VS Yes |
| Diabetes | Admission diagnoses | No VS Yes |
| Coronary artery disease | Admission diagnoses | No VS Yes |
| COPD/emphysema | Admission diagnoses | No VS Yes |
| Moderate or severe renal disease | Admission diagnoses | No VS Yes |
| Liver disease | Admission diagnoses | No VS Yes |
| Bacterial vaginosis | Admission diagnoses | No VS Yes |
| **Preoperative variables** |  |  |
| FIGO stage≥III | The FIGO stage was determined according to the FIGO 2014 guidelines | <III VS ≥III |
| ASA class≥III | American Society of Anesthesiologists Physical Status Classification System(2) | <III VS ≥III |
| Site of Cancer |  |  |
| Cervix | According to characteristic pathological diagnosis | No VS Yes |
| Ovary/Fallopia | According to characteristic pathological diagnosis | No VS Yes |
| Tube/Peritoneum Uterus | According to characteristic pathological diagnosis | No VS Yes |
| Barthel Index | The Barthel Index was used to assess the patients’ functional capacity.  Proposed guidelines for interpreting Barthel scores are that scores of 0-20 indicate “total” dependency, 21-60 indicate “severe” dependency, 61-90 indicate “moderate” dependency, and 91-99 indicates “slight” dependency. Most studies apply the 60/61 cutting point. (3) | Independent VS Partially/Totally dependent |
| MFS score | The Morse fall scale assessed the risk of falling for hospital in-patients:  No Risk (0-24); Low Risk (25-50); High Risk (≥51) (4) | No Risk VS Low /High Risk |
| Preoperative steroid use | Regular administration of hydrocortisone, methylprednisolone, or prednisolone before operation | No VS Yes |
| Median laboratory values |  |  |
| Glucose | Fasting blood glucose within 7 days before surgery | in units of mg/dL |
| Albumin | Albumin levels within 7 days before surgery | >3.0 g/dL VS ≤3.0 g/dL |
| ALT | Aminotransferase levels within 7 days before surgery | ≤40 U/L VS >40 U/L |
| Total bilirubin | Total bilirubin levels within 7 days before surgery | ＜1.1 mg/dL VS ≥1.1 mg/dL |
| Platelet count | Platelet count within 7 days before surgery | ≤350×10^9^/L VS >350×10^9^/L |
| Hematocrit | Hematocrit within 7 days before surgery | ≥36% VS <36% |
| TLC | Total lymphocyte count within 7 days before surgery | ≥0.8×10^9^ /L VS <0.8×10^9^ /L |
| WBC | White blood cell count within 7 days before surgery | ≤10×10^9^ /L VS >10×10^9^ /L |
| Preoperative hair removal | Surgical preparation for hair removal and body surface cleaning at the corresponding site of surgery | No VS Yes |
| Preoperative LOS | The time interval between hospital admission and operation | in units of day |
| Antibiotic prophylaxis within 0.5-1 hour before operation | Antibiotic prophylaxis was administered within 0.5-1 hour before the incision | No VS Yes |
| **Intraoperative variables** |  |  |
| Surgical approach | Laparotomy or Laparoscopy | Laparotomy VS Laparoscopy |
| Operative time | The time from skin incision to time of closure | in units of min |
| Estimated blood loss | The total blood loss during the surgery, which was evaluated during the surgery | in units of mL |
| Blood transfusion | Any non-autologous transfusion of whole or packed RBCs | No VS Yes |
| Emergent surgery | Surgery within 24 h of admission to the hospital | No VS Yes |

Abbreviations: BMI, body mass index; COPD, chronic obstructive pulmonary disease; FIGO, International Federation of Gynecology and Obstetrics; ASA, American Society of Anaesthesiology; MFS, Morse Fall Scale; ALT, alanine aminotransferase; TLC, total lymphocyte count; WBC, white cell count; LOS, length of stay ;.

**References**

1. Charlson M, Szatrowski TP, Peterson J, Gold J. Validation of a combined comorbidity index. *J Clin Epidemiol* (1994) **47**:1245–1251. doi:10.1016/0895-4356(94)90129-5

2. Horvath B, Kloesel B, Todd MM, Cole DJ, Prielipp RC. The Evolution, Current Value, and Future of the American Society of Anesthesiologists Physical Status Classification System. *Anesthesiology* (2021) **135**:904–919. doi:10.1097/ALN.0000000000003947

3. Mahoney FI, Barthel DW. FUNCTIONAL EVALUATION: THE BARTHEL INDEX. *Md State Med J* (1965) **14**:61–65.

4. O’Connell B, Myers H. The sensitivity and specificity of the Morse Fall Scale in an acute care setting. *J Clin Nurs* (2002) **11**:134–136. doi:10.1046/j.1365-2702.2002.00578.x
