# Supplementary material for: Development and Validation of a Nomogram Based on Geriatric Nutritional Risk Index to Predict Surgical Site Infection Among Gynecologic Oncology Patients
Source: Front Nutr. 2022 Apr 27;9:864761. doi: 10.3389/fnut.2022.864761 (PMC9097080; doi:10.3389/fnut.2022.864761)
Supplement: Supplementary file 5 [file Table_2.DOCX]

**Table S2. Criteria for defining surgical site infections.**

| **Superficial incisional surgical site infection ^a^**  Infection involves only skin or subcutaneous tissue of the incision and at least one of the following:  1. Purulent drainage, with or without laboratory confirmation, from the superficial incision.  2. Organisms isolated from aseptically obtained culture of fluid or tissue from the superficial incision.  3. At least one of the following signs or symptoms of infection: pain or tenderness, localized swelling, redness, or heat and superficial incision is deliberately opened by surgeon, unless incision is culture-negative. |
| --- |
| **Deep incisional surgical site infection** ^a^  Infection involves deep soft tissues (e.g. fascial and muscle layers) of the incision *and* at least *one* of the following:  1. Purulent drainage from the deep incision but not from the organ/space component of the surgical site.  2. A deep incision spontaneously dehisces or is deliberately opened by a surgeon when the patient has at least one of the following signs or symptoms: fever (>38°C), localized pain, or tenderness, unless site is culture-negative.  3. An abscess or other evidence of infection involving the deep incision is found on direct examination, during reoperation, or by histopathologic or radiologic examination. |
| **Organ/space surgical site infection ^a^**  Infection involves any part of the anatomy (e.g. organs or spaces), other than the incision, which was opened or manipulated during an operation and at least one of the following:  1. Purulent drainage from a drain that is placed through a stab wound into the organ/space.  2. Organisms isolated from an aseptically obtained culture or fluid or tissue in the organ/space.  3. An abscess or other evidence of infection involving the organ/space that is found on direct examination, during reoperation, or by histopathologic or radiologic examination. |

^a^ Diagnosis by the surgeon or attending physician also meets the criteria for each type of surgical site infection.
