# Supplementary material for: Development and Validation of a Nomogram Based on Geriatric Nutritional Risk Index to Predict Surgical Site Infection Among Gynecologic Oncology Patients
Source: Front Nutr. 2022 Apr 27;9:864761. doi: 10.3389/fnut.2022.864761 (PMC9097080; doi:10.3389/fnut.2022.864761)
Supplement: Supplementary file 6 [file Table_3.DOCX]

**Table S3. Baseline characteristics of patients in development cohort used for nomogram construction: pre-imputation and post-imputation.**

|  | **Development cohort (N=1221)** | | |
| --- | --- | --- | --- |
|  | **pre-imputation** | **post-imputation** | ***P* value** |
| **General information** |  |  |  |
| Age (y), median (IQR) | 50.0 (18.0) | 50.0 (18.0) |  |
| BMI (kg/m^2^), median (IQR) | 23.1 (3.9) | 23.1 (3.9) |  |
| Season of admission, n(%) |  |  |  |
| Spring | 229 (18.8) | 229 (18.8) |  |
| Summer | 271 (22.2) | 271 (22.2) |  |
| Fall | 404 (33.1) | 404 (33.1) |  |
| Winter | 317 (26.0) | 317 (26.0) |  |
| Surgical history in recent three months, n (%) |  |  |  |
| No | 1167 (96.0) | 1172 (96.0) |  |
| Yes | 49 (4.0) | 49 (4.0) |  |
| Hypertension, n (%) |  |  |  |
| No | 905 (74.1) | 905 (74.1) |  |
| Yes | 316 (25.9) | 316 (25.9) |  |
| Diabetes, n (%) |  |  |  |
| No | 1072 (87.8) | 1072 (87.8) |  |
| Yes | 149 (12.2) | 149 (12.2) |  |
| Coronary artery disease, n (%) |  |  |  |
| No | 1198 (98.1) | 1198 (98.1) |  |
| Yes | 23 (1.9) | 23 (1.9) |  |
| COPD/emphysema, n (%) |  |  |  |
| No | 1206 (98.8) | 1206 (98.8) |  |
| Yes | 15 (1.2) | 15 (1.2) |  |
| Moderate or severe renal disease, n (%) |  |  |  |
| No | 1186 (97.1) | 1186 (97.1) |  |
| Yes | 35 (2.9) | 35 (2.9%) |  |
| Liver disease, n (%) |  |  |  |
| No | 923 (75.6) | 923 (75.6) |  |
| Yes | 298 (24.4) | 298 (24.4) |  |
| Bacterial vaginosis, n (%) |  |  |  |
| No | 1177 (96.4) | 1177 (96.4) |  |
| Yes | 44 (3.6) | 44 (3.6) |  |
| aCCI (points), median (IQR) | 1.0 (4.0) | 1.0 (4.0) |  |
| **Preoperative variables** |  |  |  |
| FIGO stage, n (%) |  |  | 0.617 |
| < III | 943 (77.2) | 1123 (92.0) |  |
| ≥III | 76 (6.2) | 98 (8.0) |  |
| Missing | 203（16.6） |  |  |
| ASA class, n (%) |  |  | 0.993 |
| < III | 1157 (94.7) | 1159 (94.9) |  |
| ≥III | 62 (5.1) | 62 (5.1) |  |
| Missing | 2（0.2） |  |  |
| Site of Cancer, n (%) |  |  |  |
| Cervix | 835 (68.4) | 835 (68.4) |  |
| Ovary/Fallopia | 148 (12.1) | 148 (12.1) |  |
| Tube/Peritoneum Uterus | 238 (19.5) | 238 (19.5) |  |
| Barthel Index, n (%) |  |  |  |
| Independent | 1133 (92.8) | 1133 (92.8) |  |
| Partially/ Totally dependent | 88 (7.2) | 88 (7.2) |  |
| Morse Fall Scale, n (%) |  |  |  |
| No Risk | 930 (76.2) | 930 (76.2) |  |
| Low /High Risk | 291 (23.8) | 291 (23.8) |  |
| Preoperative steroid use , n (%) |  |  |  |
| No | 1207 (98.9) | 1207 (98.9) |  |
| Yes | 14 (1.1) | 14 (1.1) |  |
| Laboratory values, median (IQR) |  |  |  |
| Glucose (mg/dL) | 5.1 (1) | 5.1 (1) |  |
| Albumin (g/dL) | 40.5 (5.3) | 40.5 (5.3) |  |
| ALT(U/L) | 16.0 (13.0) | 16.0 (13.6) | 0.637 |
| Missing | 50（4.1） |  |  |
| Total bilirubin(mg/dL) | 10.4 (5.5) | 10.4 (5.5) | 0.740 |
| Missing | 69（5.7） |  |  |
| Platelet count(10^9^/L) | 225.0 (75.0) | 225.0 (75.0) |  |
| Hematocrit(%) | 37.9 (5.3) | 37.9 (5.3) |  |
| TLC(10^9^ /L) | 37.9 (5.3) | 37.9 (5.3) |  |
| WBC(10^9^ /L) | 5.7 (2.2) | 5.7 (2.2) |  |
| Preoperative hair removal, n (%) |  |  |  |
| No | 38 (3.1) | 38 (3.1) |  |
| Yes | 1183 (96.9) | 1183 (96.9) |  |
| Preoperative LOS (d), median (IQR) | 2.0 (2.0-4.0) | 2.0 (2.0-4.0) |  |
| Antibiotic prophylaxis within 0.5-1 hour before operation, n (%) |  |  |  |
| No | 353 (28.9) | 353 (28.9) |  |
| Yes | 868 (71.1) | 868 (71.1) |  |
| MSCS, n (%) |  |  |  |
| ≤2 | 1178 (96.5) | 1178 (96.5) |  |
| >2 | 43 (3.5) | 43 (3.5) |  |
| **Intraoperative variables** |  |  |  |
| Surgical approach, n (%) |  |  |  |
| Laparotomy | 642 (52.6) | 642 (52.6) |  |
| Laparoscopy | 579 (47.4) | 579 (47.4) |  |
| Operative time (min), median (IQR) | 90.0 (136.0) | 90.0 (136.0) |  |
| Estimated blood loss (mL), median (IQR) | 50.0 (90.0) | 50.0 (90.0) |  |
| Blood transfusion, n (%) |  |  |  |
| No | 1161 (95.1) | 1161 (95.1) |  |
| Yes | 60 (4.9) | 60 (4.9) |  |
| Emergent surgery, n (%) |  |  | 0.892 |
| No | 1022 (83.6) | 1028 (84.2) |  |
| Yes | 189 (1.6) | 193 (15.8) |  |
| Missing | 10（0.8） |  |  |
| NNIS risk index, n (%) |  |  | 1.000 |
| 0 | 933 (76.4) | 935 (76.6) |  |
| 1 | 263 (21.5) | 263 (21.5) |  |
| 2 | 23 (1.9) | 23 (1.9) |  |
| Missing | 2（0.2） |  |  |
| **Nutrition risk screening tool** |  |  |  |
| GNRI(points), median (IQR) | 102.3 (8.3) | 102.3 (8.3) |  |

Abbreviations: BMI, body mass index; COPD, chronic obstructive pulmonary disease; aCCI, age-adjusted Charlson comorbidity index; FIGO, International Federation of Gynecology and Obstetrics; ASA, American Society of Anaesthesiology; ALT, alanine aminotransferase; TLC, total lymphocyte count; WBC, white cell count; LOS, length of stay ; MSCS, modified surgical complexity score; NNIS, National Nosocomial Infection Surveillance ; GNRI, geriatric nutritional risk index.
