# Supplementary material for: Development and Validation of a Nomogram Based on Geriatric Nutritional Risk Index to Predict Surgical Site Infection Among Gynecologic Oncology Patients
Source: Front Nutr. 2022 Apr 27;9:864761. doi: 10.3389/fnut.2022.864761 (PMC9097080; doi:10.3389/fnut.2022.864761)
Supplement: Supplementary file 7 [file Table_4.DOCX]

**Table S4. Cut-off point of preoperative LOS,operative time and estimated blood loss after adjustment of the effect modifier**

| Type of model |  | Preoperative LOS^†^ | Operative time^‡^ | Estimated blood loss^§^ |
| --- | --- | --- | --- | --- |
| One-line linear regression model | OR (95%CI) | 1.13 (1.05, 1.22) | 1.00 (1.00, 1.01) | 1.00 (1.00, 1.00) |
| Two-piecewise linear model | Cut-off point | 8 | 145 | 40 |
|  | OR1 (95%CI) | 1.06 (0.92, 1.22) 0.418 | 1.02 (1.01, 1.03)  <0.001 | 1.04 (1.01, 1.07) |
|  | OR2 (95%CI) | 1.23(1.05, 1.43) 0.009 | 0.99(0.99, 1.00) | 1.00 (1.00, 1.00) |
|  | OR2/OR1 (95%CI) | 1.16 (0.90, 1.49)  0.257 | 0.98 (0.96, 0.99)  <0.001 | 0.96 (0.93, 0.99) |
|  | Logarithmic likelihood ratio test | 0.255 | <0.001 | 0.002 |

Abbreviations: OR, odds ratio;CI, confidence interval; LOS, length of stay.

^†^Adjusted for all variables listed in Table 1 except preoperative LOS.

^‡^Adjusted for all variables listed in Table 1 except operative time.

^§^Adjusted for all variables listed in Table 1 except estimated blood loss.
